# Supplementary material for: MetaRibo-Seq measures translation in microbiomes
Source: Nat Commun. 2020 Jun 29;11:3268. doi: 10.1038/s41467-020-17081-z (PMC7324362; doi:10.1038/s41467-020-17081-z)
Supplement: Supplementary file 10 — Supplementary Data 7 [file 41467_2020_17081_MOESM10_ESM.zip › File2/Confidence_High_Taxonomy/158344_out.krona.html]

Javascript must be enabled to view this page.

members
magnitude
magnitudeUnassigned
count
unassigned
taxon
rank

158344\_out

29

2
29
superkingdom

phylum
29
1239

class
29
186801

order
186802
3
29

SRS075716\_contig\_number\_contig-100\_12942.12943SRS146764\_contig\_number\_contig-100\_5267.5267SRS148784\_contig\_number\_contig-100\_12112.120424

31979
family
20


SRS017307\_contig\_number\_13036
1
20
1485
genus

2293035

SRS014613\_contig\_number\_17449SRS023526\_contig\_number\_23577SRS049959\_contig\_number\_19081SRS098571\_contig\_number\_66628SRS104311\_contig\_number\_32409SRS142781\_contig\_number\_11002SRS143780\_contig\_number\_12814SRS146812\_contig\_number\_43408
8
species

1262836
species
1

SRS075984\_contig\_number\_18727


SRS015663\_contig\_number\_2988SRS016989\_contig\_number\_contig-100\_2913.39435SRS018623\_contig\_number\_264SRS054956\_contig\_number\_1288SRS063040\_contig\_number\_22834SRS1041038\_contig\_number\_1494SRS1041144\_contig\_number\_469SRS1055034\_contig\_number\_4676SRS143523\_contig\_number\_13639
species
9
1896983

759821

SRS097889\_contig\_number\_15852
species
1

186803
6
family

species
6

SRS016095\_contig\_number\_contig-100\_122.159953SRS019068\_contig\_number\_64555SRS024435\_contig\_number\_40812SRS075078\_contig\_number\_contig-100\_55.174538SRS075773\_contig\_number\_14600SRS144135\_contig\_number\_contig-100\_2618.149928
1898203
